# Cobalt-based Catalysts for Ammonia Decomposition

**DOI:** 10.3390/ma6062400

**Published:** 2013-06-10

**Authors:** Zofia Lendzion-Bielun, Urszula Narkiewicz, Walerian Arabczyk

**Affiliations:** Institute of Chemical and Environment Engineering, West Pomeranian University of Technology, Szczecin, Pulaskiego 10, 70-322 Szczecin, Poland; E-Mails: un@zut.edu.pl (U.N.); arab@zut.edu.pl (W.A.)

**Keywords:** ammonia decomposition, cobalt catalyst, activity

## Abstract

An effect of promoters such as calcium, aluminium, and potassium oxides and also addition of chromium and manganese on the structure of cobalt catalysts was examined. Studies of the catalytic ammonia decomposition over the cobalt catalysts are presented. The studies of the ammonia decomposition were carried out for various ammonia-hydrogen mixtures in which ammonia concentration varied in the range from 10% to 100%. Co(0) catalyst, promoted by oxides of aluminium, calcium, and potassium, showed the highest activity in the ammonia decomposition reaction. Contrary to expectations, it was found that chromium and manganese addition into the catalysts decreased their activity.

## 1. Introduction

In traditional hydrogen production methods, such as natural gas steam reforming, catalytic biomass gasification, and coal gasification, the processes are connected with generation of by-products, for example carbon oxides. The by-products may be harmful not only for the environment but also for hydrogen cells. Ammonia is a good source of pure hydrogen. In comparison with hydrogen, it is easy to store and to transport, because it is liquid at the temperature of 25 °C under the pressure of 8 atm. Nitrogen and hydrogen are the unique products of the ammonia decomposition. Ruthenium supported on a carbon carrier is the most active catalyst in the ammonia decomposition reaction so far. Nevertheless, ruthenium is very expensive and its availability is limited, which inhibits its widespread use in industry [1]. Catalytic decomposition of ammonia over transition metals is an alternative for systems based on noble metals such as Ru, Pt, Pd, and Rh. Transition metals, Fe [2,3,4,5,6,7,8], Ni [5,6,9], and Co [5,7], and also their nitrides and carbides are being intensively investigated as potential catalytic systems for the ammonia decomposition reaction.

Sorensen *et al.* [7] performed investigations in which four non-promoted catalysts, Fe, Ru, Co, and Pd, were compared one another. At process conditions (575 and 650 °C, a gas mixture containing 50 vol % of ammonia and 50 vol % of argon), they found that cobalt was the most effective catalyst in the ammonia decomposition reaction. At both temperatures reaction rates, given as mole of H_2_/mole of metal/s, were five times higher for cobalt than those for iron.

Zhang *et al.* [6] found that commercial carbon nanotubes containing Co nanoparticles were highly active in the ammonia decomposition reaction. At 700 °C and under the flow of 2000 cm^3^·g^−1^·h^−1^, complete conversion of ammonia was almost reached over the carbon nanotubes based catalyst containing 4.1% of Co.

In our previous work, we compared a cobalt-based catalyst promoted with calcium, aluminium, and potassium with an industrial iron catalyst [10]. A composition of the prepared cobalt catalyst corresponded to the industrial iron catalyst for the ammonia synthesis (similar content of structural promoters), however a slight difference in a specific surface area working to the advantage of the cobalt catalyst was observed. Under conditions of the experiments (temperatures from 400 to 550 °C, an atmospheric pressure, an ammonia concentration in the mixture with hydrogen about 6 vol %), it was found that the cobalt catalyst was more effective than the iron one in the ammonia decomposition reaction. An apparent activation energy of the ammonia decomposition was determined: 111 kJ/mol and 138 kJ/mol for the cobalt and the iron catalyst, respectively. However, it should be taken into account, that the described investigations concerned kinetics measurements and they were carried out for low ammonia concentrations. Conditions of these investigations differed markedly from those required for fuel cell applications.

On the basis of the model developed for assessing the active surface of the iron catalyst for the ammonia synthesis [11], the theoretical assumptions were made in order to prepare a cobalt catalyst being active in the ammonia decomposition. According to the model [11] the iron surface is wetted by oxides of promoters, which form a two-layer structure. The first layer, in direct contact with iron, is composed of oxygen atoms. Atoms of promoter are located above oxygen atoms. In the case of the triple promoted catalyst, the surface of the catalyst is dominantly covered by a two-dimension Fe–O–K layer, whereas the rest of the promoters are located in a three-dimension bridge structure, which are bound by single iron crystallites. The basic assumption of the model is the existence of the chemical equilibrium between iron crystallites, promoters wetting the iron surface and promoters that form three-dimension structures (oxygen bridges) connecting individual iron crystallites. Not covered iron atoms (–O–M) are the sites where N_2_ molecules can be adsorbed and hydrogenated to obtain ammonia. The surface layer structure is determined by the character of elements occurring on the surface. In accordance with the model, a specific surface area of a catalyst is proportional to amount of iron atoms located on the surface and to their bond energy with oxygen. A catalyst activity is proportional to the number of free, uncovered iron atoms on the catalyst surface.

The idea of a new catalyst preparation was to replace iron atoms by cobalt atoms. For cobalt the dissociative adsorption heat of nitrogen, 134 kJ/mol [12], is lower, than that of iron 205 kJ/mol [12]. Co–O bond enthalpy is lower than Fe–O bond enthalpy, therefore according to the model, specific surface area of pure cobalt is lower. However, cobalt may be expected to be more resistant against poisoning.

One can assume that introducing into the system elements bonding oxygen strongly than cobalt, for example manganese and/or chromium, an increase in the specific surface area of the catalysts is expected.

In this work, preparation and characterization of cobalt catalysts for the ammonia decomposition are presented. The influence of the composition of hydrogen-ammonia gas mixtures on the kinetics of the ammonia decomposition was examined.

## 2. Experimental

### 2.1. Preparation of Catalysts

Cobalt(II) hydroxide was precipitated with an NH_4_OH solution from a water solution of cobalt(II) nitrate (Co(NO_3_)_2_·6H_2_O, Merck). During the precipitation, the reaction was being controlled continuously to maintain the pH at a constant value, (pH = 8.5). The obtained suspension was washed with demineralized water to remove nitrate ions; then dried at 105 °C for 12 h and calcined at 200 °C for 2 h. In the next stage promoters were introduced by wet co-impregnation with adequate water solutions of calcium, potassium, and aluminium nitrates. In the same way, chromium and manganese were introduced into the catalysts. Manganese acetate and chromium nitrate were used to prepare a solution for the impregnation. The impregnation was carried out in a vacuum evaporator (Rotavapor R-210, BUCHI). Concentrations of the respective salts were calculated to reach established contents of the promoters in the catalyst: CaO—2.6%, Al_2_O_3_—3.0%, K_2_O—0.6% and from 0.2% to 0.4% MnO_2_ or Cr_2_O_3_—from 0.2% to 0.4%. After the impregnation, the calcination process was carried out for 4 h at 200 °C and for 2 h at 500 °C.

### 2.2. Characterization of Catalysts

A quantitative analysis of elements in the catalysts was determined with atomic emission spectroscopy with inductively coupled plasma ICP-OES (spectrometer Optima 5300 DV, Perkin Elmer) and using scanning an electron microscope SEM (DSM 962, Zeiss) with an X-ray analyser EDS (detector X-flash 4010, Bruker).

A phase composition and an average crystallite size of cobalt oxide crystallites were determined by using an X-ray diffraction method XRD (X^’^Pert PRO Philips diffractometer with CuK_α_ radiation). Line (440) of Co_3_O_4_ and Scherrer’s equation were used in order to calculate an average size of crystallites.

Specific surface area (BET) of the oxidized form of the catalysts was determined by nitrogen adsorption-desorption measurements at 77 K using a Micromeritics ASAP 2010 instrument. The reduction behaviours of samples were examined by a temperature-programmed reduction with H_2_ (H_2_-TPR) technique using a Micromeritics AutoChem 2920 instrument. The same equipment was used to determine specific surface area of the catalysts after reduction at 600 °C and heating in hydrogen atmosphere.

### 2.3. Catalytic Tests

Catalytic tests were carried out in a differential quartz reactor connected to a gas analyser and a thermogravimetric mass changes recorder. The samples of the oxidized form of the catalysts, approximately 0.5 g, were used in tests. They were placed in the reactor as a monolayer of grains (1–1.2 mm) in a platinum basket. Blank tests were performed to confirm a lack of influence of platinum on the reaction; they indicated that the ammonia conversion in the empty reactor was less than 1%. Prior to kinetics measurements of the ammonia decomposition reaction, the catalysts were reduced with hydrogen, purity of 99.999%, at 600 °C and a flow rate of 200 cm^3^ min^−1^. When the reduction process had been finished, the catalysts were heated for 3 h in hydrogen atmosphere, at the same temperature. For kinetics tests, pure ammonia (99.998%) was used; its flow rate varied in the range from 30 to 200 cm^3^/min. In order to determine the influence of hydrogen on the kinetics of the ammonia decomposition reaction various ammonia-hydrogen mixtures were used, with the ammonia content from 10 to 100 vol %. Gaseous mixture composition was varied, but GHSV (gas hourly space velocity) remained unchanged, at the level of 24,000 cm^3^·g^−1^·h. Gas flow rates were being controlled by mass flow controllers. Activities of the catalysts were examined under atmospheric pressure, at 500 and 550 °C.

On the basis of balance of reaction system, in which the reaction NH_3_ = 1.5H_2_ + 0.5N_2_ takes place, one obtains [3]:

*F*_NH3_ = *F*^0^_NH3_ − *α*_NH3_*F*^0^_NH3_ (1)

*F*_H2_ = *F*^0^_H2_ − 1.5 *α*_NH3_*F*^0^_NH3_ (2)

*F*_N2_ = 0.5 *α*_NH3_*F*^0^_NH3_ (3)

*F*^0^ = *F*^0^_NH3_ + *F*^0^_H2_ (4)

*F*_out_ = *F*_NH3_ + *F*_H2_ + *F*_N2_ (5)

After transformations of Equations (1–5) the expression describing the conversion degree of ammonia was obtained as follows:

(6) $$\alpha_{\text{NH3}}=\frac{X_{\text{H2}}F^{0}-F_{\text{H2}}^{\text{0}}}{F_{\text{NH3}}^{\text{0}}(1.5-X_{\text{H2}})}$$

Where: *F*^0^ is total gases flow at the reactor inlet mol·s^−1^, *F*^0^_H2_ and *F*^0^_NH3_ are hydrogen and ammonia molar flows at the reactor inlet, mol·s^−1^, *X*_H2_ is mole fraction of hydrogen in the reactor in stationary state.

On the basis of the ammonia conversion degree under given conditions of temperature and the ammonia flow rate at the reactor inlet, the rate of the ammonia decomposition related to mass of the catalyst was calculated from an Equation (7).

(7) $${\text{r}}_{\text{decomp}}=\alpha_{\text{NH3}}F_{\text{NH3}}^{0}/{\text{m}}_{\text{cat}}$$

where: m_cat_ is mass of catalyst, g.

## 3. Results and discussion

As the result of the precipitation of cobalt hydroxide and then the calcination at 200 °C for 2 h, a catalyst precursor, cobalt (II, III) oxide, was obtained,. Such obtained cobalt oxide (Co), with specific surface area *ca.* 64 m^2^/g, was impregnated with promoter solutions.

The nomenclature of the catalysts, compositions, average crystallite sizes of Co_3_O_4_, and specific surface areas (BET) are summarized in Table 1.

**Table 1:** Chemical composition and specific surface areas of the catalysts.

catalyst	content [wt %]						d_Co3O4_	S_o_	S_600_
	Al_2_O_3_	CaO	K_2_O	Cr_2_O_3_	MnO_2_	Co_3_O_4_	(nm)	(m^2^/g)	(m^2^/g)
Co	^–^	^–^	^–^	^–^	^–^	100	11	64	4
Co(0) [13]	2.6 ^a^/2.3 ^b^	1.5 ^a^/1.7 ^b^	0.5 ^a^	^–^	^–^	95.4	23	29	13
CoMn(0.25) [13]	2.8 ^a^/2.6 ^b^	1.4 ^a^/2.4 ^b^	0.6 ^a^	^–^	0.39 ^a^	94.8	20	37	14
CoCr(0.16)	2.9 ^a^/2.7 ^b^	1.6 ^a^/1.9 ^b^	0.5 ^a^	0.23 ^a^	^–^	94.8	34	33	14
CoCr(0.28)	2.5 ^a^/2.3 ^b^	1.4 ^a^/1.2 ^b^	0.5 ^a^	0.41 ^a^/0.55 ^b^	^–^	95.2	30	39	17

^a^ calculated on the basis of the results from the ICP-OES method; ^b^ calculated from the EDX method; S_o_ is specific surface area of the oxidised form of the catalyst; S_600_ is specific surface area of the catalyst after reduction and sintering in hydrogen at 600 °C.

Numbers given in parentheses in the names of the catalysts determine the contents of chromium or manganese. Calcination at 500 °C caused a drop in specific surface area of the prepared catalysts in comparison with specific surface area of the starting cobalt oxide. A little chromium addition brought on an increase in specific surface area, Table 1. The similar phenomenon took place for catalysts with manganese addition [13]. The incorporation of small amounts of manganese into the system, the element bonding oxygen stronger than cobalt, influenced on the development of specific surface area. Specific surface areas of the catalysts decreased dramatically after reduction and calcination. It was especially observed for the cobalt catalyst without addition of promoters when its specific surface area decreased from 64 just to 4 m^2^/g after reduction and heating. An addition of promoter oxides stabilizes the surface area of the catalysts. The observed decrease in the surface area is significantly lower. In our previous works [14,15], we informed about thermostabilizing properties of calcium and aluminum.

**Figure 1:**
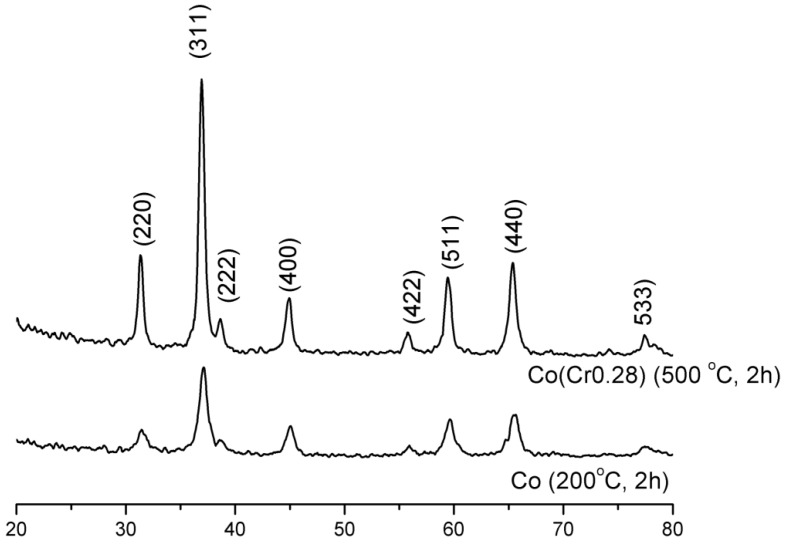
X-ray diffraction (XRD) patterns of the cobalt oxide and the cobalt catalyst.

It follows from XRD analysis that cobalt oxide Co_3_O_4_ is the main crystallographic phase in the oxidized form of the prepared catalysts, Figure 1. The example X-ray pattern of CoCr(0.28) sample was compared with the X-ray pattern of pure cobalt oxide, Figure 1. An average size of Co_3_O_4_ crystallites of pure cobalt oxide, calculated from Scherrer’s equation, was 11 nm while about 30 nm for the CoCr(0.28) catalyst. Calcination carried out at 500 °C, after the impregnation with solutions of the salt of the promoters, resulted in an increase in the average size of cobalt oxide crystallites.

**Figure 2:**
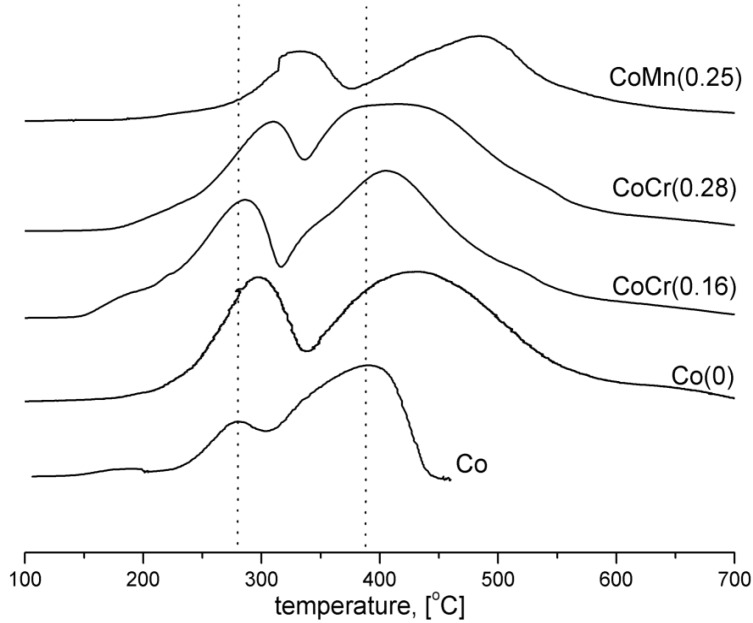
H_2_-TPR profiles of the cobalt catalysts (flow 10% H_2_/Ar, 10°/min from 100 to 700 °C).

The H_2_-TPR profiles of the catalysts are shown in Figure 2. The H_2_-TPR profile of the pure cobalt oxide (Co) illustrates two overlapping reduction peaks, at 280 °C and 380 °C, associated with the two-step reduction of Co_3_O_4_ (Co_3_O_4_ → CoO → Co) [14]. The H_2_-TPR profile of the Co(0) catalyst, with an addition of promoters, was shifted toward higher temperatures. Profile maxima for other catalysts were systematically shifted to higher temperatures than that of pure Co_3_O_4_. Reduction of CoMn(0.25) catalyst, with the addition of manganese oxide, was the slowest. An addition of chromium into CoCr(0.16) and CoCr(0.28) catalysts decreased the temperature in which the reduction began. It is in accordance with previous investigations [16].

**Figure 3:**
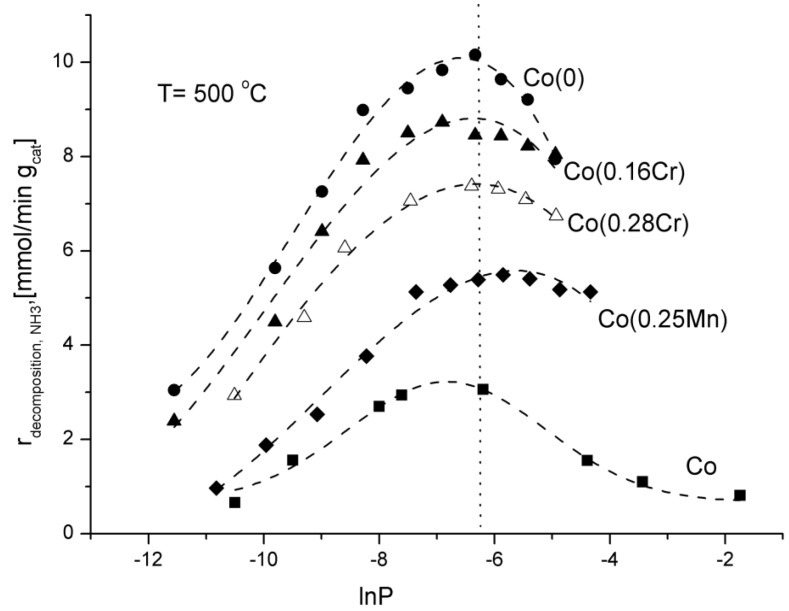
Dependence of the rate of the ammonia decomposition reaction on the logarithm of nitriding potential at the temperature of 500 °C.

Changes of the ammonia decomposition rate as a function of natural logarithm of nitriding potential, lnP, were shown in Figure 3.

The nitriding potential, P, was defined in our previous work [3] as the ratio of partial pressures of ammonia and hydrogen, P = p_NH3_/p_H2_^1.5^. This parameter has a significant impact on the ammonia decomposition kinetics. The values of partial pressures were calculated on the basis of the material balance of the reactor and hydrogen concentrations at the outlet of the reactor in stationary states.

Ammonia decomposition rate increased while potential P increased to about P = 0.0019 Pa^−0.5^ reaching the maximum value at that point. Having reached the maximum value, ammonia decomposition rate diminished while ammonia partial pressure increased in the reaction atmosphere. In the previous investigations, the same phenomenon was observed for an iron catalyst [3]. According to K. Kielbasa *et al*., a decrease in the ammonia decomposition rate is connected with an iron nitriding reaction and a formation of a new solid phase. In the case of cobalt catalysts, mass changes were not registered. That may exclude the nitriding process. After activity tests, also XRD phase analysis did not show any new phase.

It is known from scientific literature that the grain structure of Co_4_N is very similar to the structure of Co, which might easily lead to misinterpretation [17,18]. Although, there is a conjecture that reconstruction of the surface takes place by interaction between nitrogen and cobalt atoms. Such surface changes, not observed using the thermogravimetric and XRD methods, may have significant impact on the ammonia decomposition rate.

Among the investigated catalysts, the Co(0) one (promoted with calcium oxide, aluminium oxide, and potassium oxide) showed the highest activity, expressed as the ammonia decomposition rate. The lowest activity was shown by the non-promoted Co catalyst, which should be connected with a relatively low specific surface area of 4 m^2^/g after the reduction process. An addition of manganese and chromium slowed down the ammonia decomposition. The Co(0) catalyst showed the highest activity (Table 2), for pure ammonia at the reactor inlet and GHSV_NH3_ = 24,000 mL·h^−1^·g_cat_^−1^, at temperatures of 500 and 550 °C. The ammonia conversion degree was 40.1% and 50%, respectively.

**Table 2:** NH_3_ conversion degree and H_2_ formation rate over cobalt catalysts (GHSV_NH3_ = 24,000 mL·h^−1^·g_cat_^−1^, at temperatures 500 and 550°C).

catalyst	NH_3 _conversion degree (%)			H_2_ formation rate (mmol/min g_cat_)	
	500 °C	550 °C		500 °C	550 °C
Co	4.5	26.2		1.2	5.5
Co(0)	40.1	50.0		8.3	9.5
CoMn(0.25)	23.0	–		6.2	–
CoCr(0.16)	35.0	45.0		7.3	8.3
CoCr(0.28)	32.0	41.0		6.8	7.7

Adjusting the reactor feed, it is possible to reach high ammonia conversion degrees over the cobalt catalyst promoted with aluminium, calcium, and potassium oxides.

**Figure 4:**
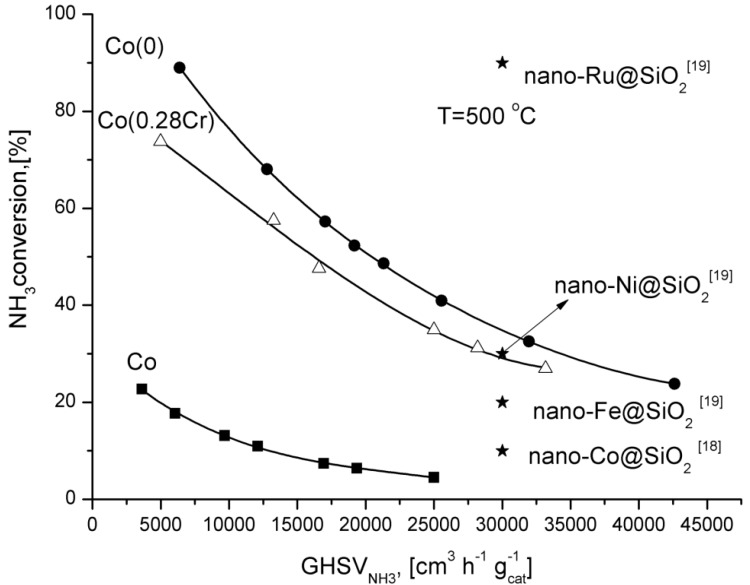
Dependence of the ammonia conversion degree at 500 °C as a function of GHSV.

For comparison, ammonia conversion degrees obtained over supported catalysts promoted with various metals are shown in Figure 4. Only the ruthenium catalyst was more active than Co(0) catalyst.

## 4. Conclusions

Cobalt oxide with a high surface area was obtained. The reduction at the temperature of 600 °C causes a sintering of pure cobalt. An addition of promoter oxides stabilizes the surface area of the catalysts. A small addition of chromium or manganese leads to an increase in the specific surface area of the cobalt catalyst. However, the presence of chromium or manganese in the catalyst decreases the activity of the examined catalysts in the ammonia decomposition reaction.

## References

[1] Zhang J., Xu H.Y., Jin X.L., Ge Q.J., Li W.Z. (2006). Highly efficient Ru/MgO catalysts for NH_3_ decomposition: Synthesis, characterization and promoter effect. Catal. Commun..

[2] Arabczyk W., Zamłynny J. (1999). Study of the ammonia decomposition over iron catalysts. Catal. Lett..

[3] Kielbasa K., Pelka R., Arabczyk W. (2010). Studies of the Kinetics of Ammonia Decomposition on Promoted Nanocrystalline Iron Using Gas Phases of Different Nitriding Degree. J. Phys. Chem. A.

[4] Kowalczyk Z., Sentek J., Jodzis S., Muhler M., Hinrichsen O. (1997). Effect of Potassium on the Kinetics of Ammonia Synthesis and Decomposition over Fused Iron Catalyst at Atmospheric Pressure. J. Catal..

[5] Duan X., Ji J., Qian G., Fan Ch., Zhu Y., Zhou X., Chen D. (2012). Ammonia decomposition on Fe(1 1 0), Co(1 1 1) and Ni(1 1 1) surfaces: A density functional theory study. J. Mol. Catal. A Chem..

[6] Zhang J., Comotti M., Schuth F., Schlogl R., Su D.S. (2007). Commercial Fe- or Co-containing carbon nanotubes as catalysts for NH_3_ decomposition. Chem. Commun..

[7] Sorensen R.Z., Nielsen L.J.E., Jensen S., Hansen O., Johannessen T., Quaade U., Christensen C.H. (2005). Catalytic ammonia decomposition: Miniaturized production of CO*_x_*-free hydrogen for fuel cells. Catal. Commun..

[8] Lanzani G., Laasonen K. (2010). NH_3_ adsorption and dissociation on a nanosized iron cluster. Int. J. Hydrog. Energy.

[9] Chellappa A.S., Fischer C.M., Thomson W.J. (2002). Ammonia decomposition kinetics over Ni-Pt/Al_2_O_3_ for PEM fuel cell applications. Appl. Catal. A Gen..

[10] Lendzion-Bielun Z., Pelka R., Arabczyk W. (2009). Study of the Kinetics of Ammonia Synthesis and Decomposition on Iron and Cobalt Catalysts. Catal. Lett..

[11] Arabczyk W., Narkiewicz U., Moszyński D. (1999). Double-Layer Model of the Fused Iron Catalyst for Ammonia Synthesis. Langmuir.

[12] Aika K., Tamaru K., Nielsen A. (1995). Ammonia synthesis over non-iron catalysts and related phenomena. Ammonia, Catalysis and Manufacture.

[13] Lendzion-Bielun Z. (2012). The effect of manganese on the structural and surface properties of nanocrystalline cobalt catalyst for ammonia synthesis. Cent. Eur. J. Chem..

[14] Lendzion-Bielun Z., Jedrzejewski R., Arabczyk W. (2011). The effect of aluminium oxide on the reduction of cobalt oxide and thermostabillity of cobalt and cobalt oxide. Cent. Eur. J. Chem..

[15] Arabczyk W., Jasinska I., Lendzion-Bielun Z. (2011). Kinetics studies of recrystallization process of metallic catalysts for ammonia synthesis. Catal. Today.

[16] Boucetta Ch., Kacimi M., Ensuque A., Piquemal J.-Y., Bozon-Verduraz F., Ziyad M. (2009). Oxidative dehydrogenation of propane over chromium-loaded calcium-hydroxyapatite. Appl. Catal. A Gen..

[17] Yaoa Z., Zhua A., Chena J., Wanga X., Auc C.T., Shia Ch. (2007). Synthesis, characterization and activity of alumina-supported cobalt nitride for NO decomposition. J. Solid State Chem..

[18] Yao L.H., Li Y.X., Zhao J., Ji W.J., Au C.T. (2010). Core–shell structured nanoparticles M@SiO_2_, Al_2_O_3_, MgO; M = Fe, Co, Ni, Ru) and their application in CO*_x_*-free H_2_ production via NH_3_ decomposition. Catal. Today.

